# Metagenomic approaches in microbial ecology: an update on whole-genome and marker gene sequencing analyses

**DOI:** 10.1099/mgen.0.000409

**Published:** 2020-07-24

**Authors:** Ana Elena Pérez-Cobas, Laura Gomez-Valero, Carmen Buchrieser

**Affiliations:** ^1^​ Institut Pasteur, Biologie des Bactéries Intracellulaires, Paris, France and CNRS UMR 3525, 675724, Paris, France

**Keywords:** metagenomics, whole-genome sequencing, marker genes, sequencing data analyses

## Abstract

Metagenomics and marker gene approaches, coupled with high-throughput sequencing technologies, have revolutionized the field of microbial ecology. Metagenomics is a culture-independent method that allows the identification and characterization of organisms from all kinds of samples. Whole-genome shotgun sequencing analyses the total DNA of a chosen sample to determine the presence of micro-organisms from all domains of life and their genomic content. Importantly, the whole-genome shotgun sequencing approach reveals the genomic diversity present, but can also give insights into the functional potential of the micro-organisms identified. The marker gene approach is based on the sequencing of a specific gene region. It allows one to describe the microbial composition based on the taxonomic groups present in the sample. It is frequently used to analyse the biodiversity of microbial ecosystems. Despite its importance, the analysis of metagenomic sequencing and marker gene data is quite a challenge. Here we review the primary workflows and software used for both approaches and discuss the current challenges in the field.

## Data Statement

All supporting data, code and protocols have been provided within the article or through supplementary data files.

**Impact Statement**

The development of metagenomics and marker gene-based approaches combined with high-throughput sequencing has revolutionized the field of microbial ecology. Two approaches have been extensively used to study microbial diversity: whole-genome shotgun (WGS) and marker gene sequencing. WGS sequencing allows the characterization of whole genomes, genes and genetic features, while marker gene analysis provides an in-depth description of the diversity of specific taxonomic groups. These approaches produce millions of reads even in a single study. Thus, a significant number of methods and software have been developed in parallel to deduce meaningful information from this vast amount of data that is generated. Each microbial community varies considerably in structure and composition, making it complicated to select the optimal methodology for the analysis and interpretation of such data. A challenge in this field is choosing methods, software and databases fitting the data and the questions of the study. In this review, we provide an updated guideline for the analyses of WGS and marker gene sequencing data. We also discuss recent comparisons of the available methods, software and databases to perform those metagenomic analyses.

## Introduction

Metagenomics refers to the application of sequencing techniques to analyse the totality of the genomic material present in a sample [1]. Currently, two main methods for studying microbial communities using high-throughput sequencing are used: marker gene studies and whole-genome shotgun (WGS) metagenomics. WGS metagenomics aims to sequence all genomes existing in an environmental sample to analyse the biodiversity and the functional capabilities of the microbial community studied. As the entire genetic material of a sample is recovered, it is possible to characterize the complete diversity of a habitat, including archaea, bacteria, eukaryotes, viruses and plasmids, as well as its gene content. In contrast, marker gene analyses are based on the sequencing of a gene-specific region to reveal the diversity and composition of specific taxonomic groups present in an environmental sample. The principal marker genes used in microbial ecology are the 16S rRNA gene (to analyse the presence of archaea and bacteria) [2], the internal transcribed spacer (ITS) region (to characterize the composition of the fungal community) [3] and the 18S rRNA (to report the occurrence of eukaryotes) [4]. Since WGS metagenomics and marker gene analyses have been developed, they have set new milestones in microbial ecology. Both approaches have been used extensively to characterize microbial communities, in particular coupled with high-throughput sequencing technologies.

The main advantage of WGS metagenomics compared to marker gene sequencing is that it offers the possibility to characterize the genetic and the genomic diversity of the analysed community as well as potential and novel functions that are present in the studied community. Further, when using an appropriate sequencing depth, it is possible to assemble full genomes from metagenome data to gain insights into the ‘genomic diversity’ of microbial ecosystems and to obtain draft genomes of uncultured organisms [5–7]. Although recent approaches have been developed to classify marker gene sequences at lower taxonomic levels than the genus [8–10], it is still not possible to distinguish between genomes with similar marker gene regions, while WGS metagenomics allows us to assign taxonomy at the species and strain levels [11–13]. Moreover, in comparison to the marker gene approach, WGS metagenomics is generally less affected by the biases associated with the PCR necessary for amplifying the marker genes, such as the number of cycles used or the primers and hyper-variable regions chosen [14–16]. However, WGS metagenomics sequencing may also be affected by biases in the metagenomic output, mainly due to the use of whole-genome amplification protocols, which are applied when working with low-concentration DNA samples [17].

**Fig. 1.**
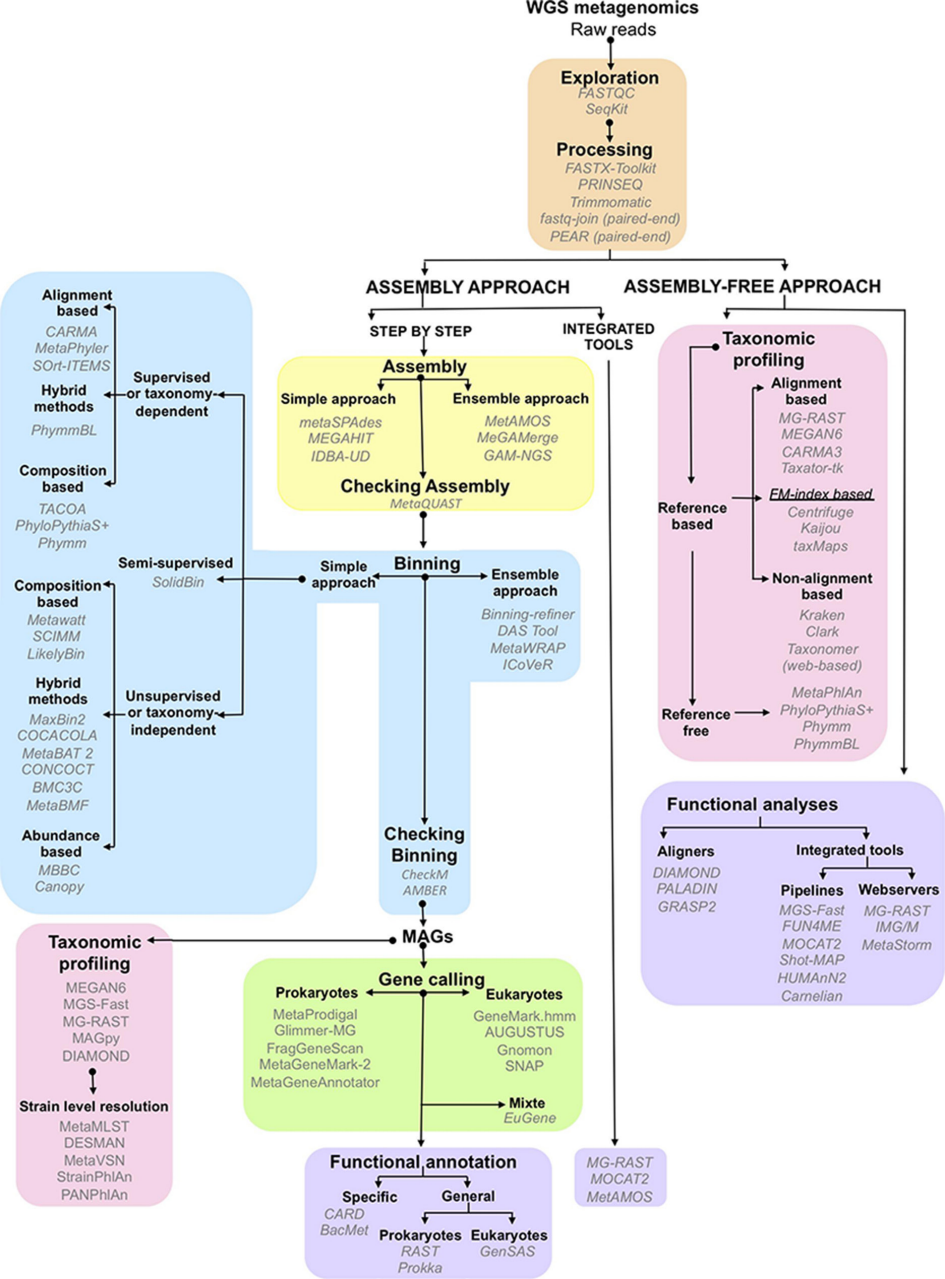
Schematic representation of the main steps necessary for the analysis of WGS metagenomics derived data. The software related to each step is shown in italics.

**Fig. 2.**
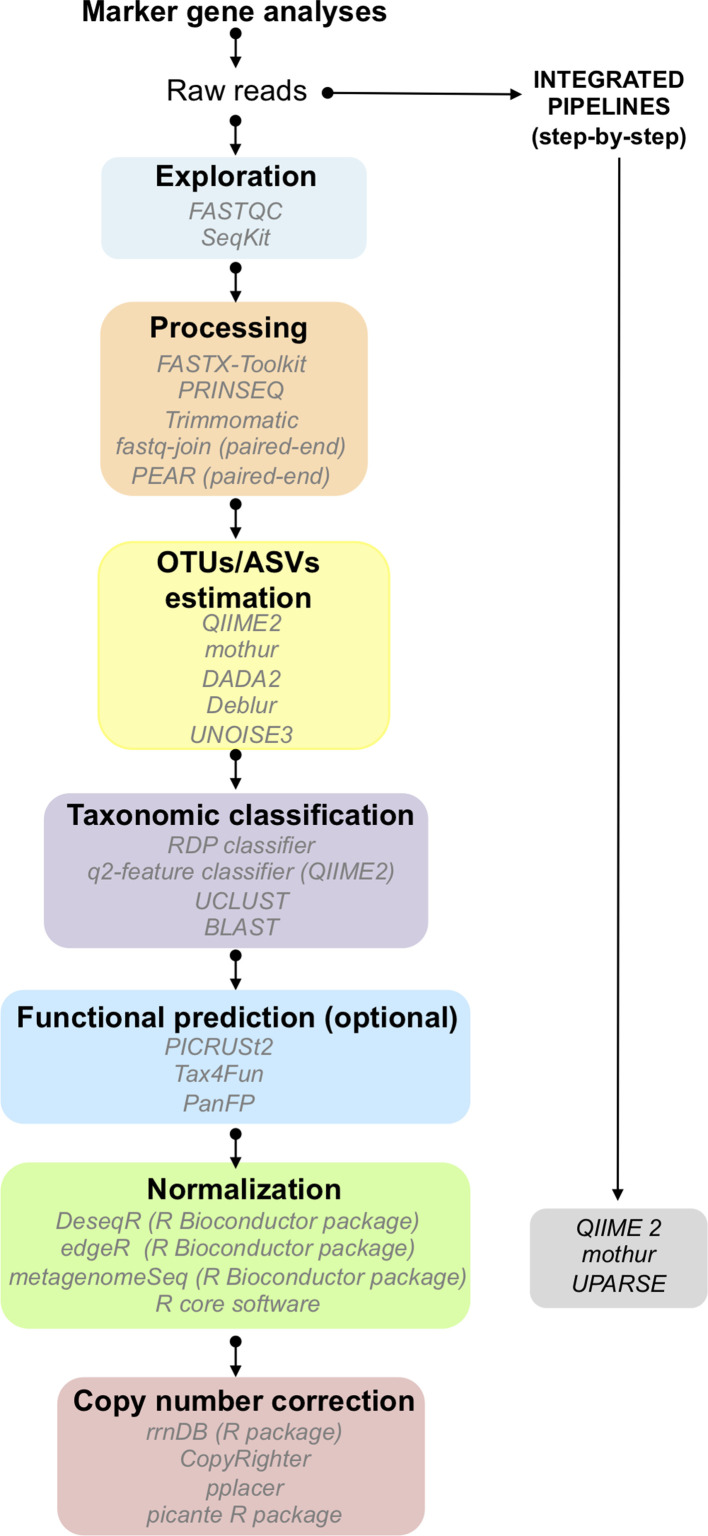
Schematic representation of the main steps necessary for the analysis of marker gene-derived data. The software related to each step is shown in italics.

Furthermore, when sequencing metagenomes, some specific chromosomal parts may be undercovered, depending on the properties of the genomic regions (GC content, secondary structures, homopolymeric regions), the sequencing depth and the chosen sequencing technology [18]. WGS metagenomics can be undertaken in habitats such as the human skin or the lungs, characterized by low biomass and high host DNA contamination [19, 20]. However, higher sequencing depth (more expensive sequencing) or host DNA depletion has to be applied, with the consequence of higher cost or bias associated with the use of additional protocols. Thus, marker gene sequencing is a more suitable option for such samples. Further, marker gene processing is generally faster, and the results are simpler to analyse and less expensive than WGS metagenomics, making it advantageous for long-term projects or studies including large numbers of samples. Both approaches have advantages and disadvantages (extensively reviewed by Knight and colleagues [21]). Thus, choosing the technique and selecting it according to the questions to be answered in the study is crucial. Here, we review the current methodology for the analysis of WGS metagenomics (overview in Fig. 1) and marker gene sequencing data (overview in Fig. 2), as well as the challenges and future perspectives, to aid in choosing the appropriate technique for different projects.

## Sequencing and quality filtering

In this review, we focus on the analysis of Illumina platform-derived data, since this sequencing technology is most commonly used in metagenomic studies. Illumina standard sequencing produces large numbers of reads (up to 1.5 Tb per run) with high accuracy (error rate ranging from 0.1–1 %), generally with a length of 150–300 bp per read. For example, the Illumina NovaSeq 6000 System can be coupled with WGS metagenomics to produce up to 6Tb per run. Other sequencing technologies, such as Oxford Nanopore MinION/GridION and Pacific Biosciences Sequel, are also used, since they can yield up to 10 Gb per run, as well as very long reads (hundreds of kb). However, the quality of MinION and PacBio sequencing is still lower than that of the Illumina system (PacBio has an error rate of 2.5 %) [22]. MinION quality scores do not follow Phred expected error rates, but the quality is lower than that for Illumina sequences [23].

Moreover, BGI NGS Platforms offer DNBSEQ technology that can be used for a range of applications. For instance, DNBSEQ-T7 is suitable for WGS metagenomics, generating 1–6 Tb of high-quality data, with read lengths of 100–150 bp. Choosing the appropriate technology among the variety of sequencing systems available will depend on the goal of the project. For example, Illumina sequencing is excellent for marker gene studies, since the analyses are based on short fragments (amplicons), and high quality is necessary to discriminate between reads. However, it is not always necessary to choose, but it may be better to apply more than one sequencing technology in a single study and combine, for example, the high-quality reads of Illumina with the long reads of MinION or Pacific Bioscience.

The aim is to distinguish natural genetic variations within the reads obtained from sequencing errors, a task that is not easily achieved. Thus, data quality control is a critical and often quite challenging step in WGS metagenomics and marker gene analyses (Figs. 1 and 2). It is crucial, as sequencing errors can lead to overestimation of the diversity in microbial community analyses and also to wrong taxonomic annotations [24]. The quality filtering and preprocessing of the reads include trimming the sequencing adapters, discarding short and low-quality reads, removing low-quality extremes, or removing reads with ‘N’ characters based on quality. Different software has been developed to achieve these tasks, such as the FASTX-Toolkit (http://hannonlab.cshl.edu/fastx_toolkit/), PRINSEQ [25] and Trimmomatic [26]. However, the determination of the required quality filtering set that needs to be applied to a specific data set is challenging. Exploratory software packages such as FASTQC [27] or SeqKit [28] are helpful as they describe the general statistics of the raw data – read length, GC content, quality score distributions, number of duplicated reads and number of N bases – but they also detect peculiarities of the data necessary for the processing. Understanding the ‘nature’ of the data is critical to perform adjusted quality control.

For marker gene studies, sequencing systems based on paired-end reads, such as the MiSeq paired-end technology (Illumina), are commonly used. After quality trimming, the paired reads need to be joined to obtain longer, higher-quality reads. Programs such as fastq-join [29] or PEAR [30] can be used for that task. The fastq-join utility joins paired-end reads on the overlapping ends. It is possible to choose the maximum allowed percentage of bases that differ in the matching region and also the minimum number of bases that must overlap for reads to be joined [29]. Similarly, PEAR joins the paired-end reads and allows us to define different parameters, such as the minimum and maximum length of output sequences or the maximum proportion of uncalled bases. Further, with PEAR it is possible to use a minimum quality score for trimming the reads while joining them [30].

Quality filtering of the data is necessary for WGS metagenomics and marker gene sequencing. Only with correctly filtered and high-quality data will analyses produce correct and meaningful estimates of the microbial diversity of the community.

## WGS metagenomics

Shotgun metagenomics is the untargeted (‘shotgun’) sequencing of all (‘meta’) microbial genomes (‘genomics’) present in a sample [31]. It can be used to profile the taxonomic composition and the functional potential of microbial communities and to recover whole-genome sequences. Although it is possible to analyse sequence data without assembly, most analyses can be improved by constructing longer, more contiguous sequences (contigs). Therefore, in the next section, we focus mainly on the steps that must be taken to obtain metagenome-assembled genomes (MAGs) and extract their functional potential in the best way.

### From reads to assembly

Assembly is the process of reconstructing *in silico* the original genome sequence from the sequenced, smaller fragments. One can either perform *de novo* assembly, by joining sequenced fragments to generate contigs without using a previously sequenced reference genome or carrying out a ‘comparative assembly’, by using a previously sequenced, closely related organism to guide the assembly. The first approach is challenging, and thus several heuristic-based strategies have been developed to solve the problem. The most used strategies (also called paradigms) are greedy, overlap–layout–consensus (OLC), or De Bruijn graph methods [32]. Greedy is an algorithm that always takes the best immediate or local solution. Hence, based on Greedy, reads that overlap the best are merged (recursively) until there are no more reads that can be joined. Since the best local solution is not considering the global possibilities, the risk is to get blocked or to provide an incorrect final assembly. Although very simple to implement, due to the disadvantages inherent to the method, Greedy has not become a popular assembly solution. In the case of OLC, all reads are compared pairwise to construct overlaps. Then overlaps are combined into a graph where nodes are reads and edges are overlaps between them. The aim is to find junctions between the overlaps even if the reads contain errors to reconstruct longer contigs. This strategy was used for the first human genome reconstruction [33]. Although it is valid, it has a high error rate, and it does not work appropriately for short reads. Thus its use has declined, but due to the re-emergence of long-read assemblies such as the Pacific Bioscience or Oxford Nanopore technologies, it has re-emerged [34]. However, Pacific Bioscience and Oxford Nanopore, which provide long but error-prone reads, are still too expensive to be applied for metagenomic studies. Furthermore, OLC is computationally demanding, a problem that the third paradigm, the De Bruijn graph method, solves better by avoiding pairwise comparisons of all reads. Instead, the De Brujin method uses substrings of fixed length (k-mers) derived from the reads and infers the overlap among them through the sharing of k-mers. To search for shared k-mers is much less computationally expensive than pairwise alignments of all reads.

Consequently, the De Brujin graph method has become the most popular assembly paradigm and is thus implemented in many assemblers, such as SPAdes, Ray Meta, or SOAPdenovo2 [35–37]. However, the De Brujin graph method is sensitive when errors in the reads are present. In addition, it is essential to choose the correct k-mer parameter; in particular, short k-mers can induce false joins when using the De Brujin paradigm. To choose the right k-mer value requires a correct estimation of parameters such as genome size, coverage, repetitive sequences, heterozygosity rate, or read error rate. For example, the presence of repeats longer than ‘k’ nucleotides can lead to a larger quantity of smaller contigs, and heterozygous regions that can complicate the graph structure and make it challenging to phase haplotypes. Consequently, several tools have been developed recently to help the user to choose an appropriate k-mer value. One example is KmerGenie [38], which provides a given set of reads with the best k-mer length for *de novo* assemblies. It can be implemented in single-k genome assemblers. More recent tools are the R package findGSE [39] or the web application GenomeScope [40]. GenomeScope is a user-friendly tool allowing the user to estimate the necessary parameters to choose the right k-mer easily. The new version of GenomeScope 2 is also applicable to polyploid genomes. Finally, some assemblers, such as IDBA [41], IDBA-UD [42] and subsequently SPAdes, SOAPdenovo2, or the recently developed ScalaDBG [43], have implemented innovative ways to deal with the choice of the best k-mer by using a multi-k-mer approach.

Genome assembly of one organism is already a complicated step. However, it is even more challenging in metagenomics, since this requires the simultaneous assembly of many genomes contained in the analysed sample. Most of the assemblers used are previously developed single-genome assemblers that have been adapted to metagenomic samples: MetaVelvet, MetaVelvetSL, MEGAHIT, metaSPAdes, Ray Meta, IDBA-UD, or SOAPdenovo2 [36, 37, 42, 44–46]. Others have been developed specifically for metagenomic sequence analyses, e.g. Minia [47], an assembler based on the De Brujin paradigm that requires small amounts of memory. Another option is MaSuRCA [48], a hybrid method combining OLC and Eulerian de Bruijn graphs. It allows one to construct super reads, making it one of the first assemblers to be capable of handling a mixture of Illumina reads and longer reads from 454 and Sanger sequencing technologies together. Currently, the most used assembly programs for metagenomics are based on the De Brujin paradigm.

Here we mention only some examples, as the number of available assemblers is so large that finally, the most challenging decision for researchers is which assembler to choose. It is beyond the scope of this review to list and describe all of the methods that have been developed (for a detailed description of the most used ones, see Vollmers *et al.* [49]). Instead, we aim to provide information that will help users to choose from among the available software. Indeed, many studies have compared different assemblers using real, simulated, or hybrid data to determine which one is the best option, and the global conclusions reached by these different studies are very similar. First, a method that could be called the best does not exist since, depending on the data at hand and the research question asked, different assembly tools prove to be optimal. If the main goal is to obtain large scaffolds, the most recommended method is metaSPAdes [31, 49–53], as it captures a high degree of community diversity, even if it shows high complexity and read coverage is low. Other multi k-mer assemblers are MEGAHIT [54] and IDBA-UD [42]. MEGAHIT requires less computational resources and is more efficient than metaSPAdes, but it is biased towards low-coverage genomes [49, 50, 55]. Therefore, when the degree of captured diversity is more important than contig lengths, or computational resources become limited, MEGAHIT [54] is the most attractive option [49]. In contrast, if the main aim of the study is to represent the largest fraction of the diversity of the analysed sample accurately, then metaSPAdes should be the assembler of choice. Whereas for low-complexity databases, such as samples with low species richness, MaSuRCA [46] is the best option [51].

According to Critical Assessment of Metagenome Interpretation (CAMI), a community-driven initiative for the critical assessment of metagenome interpretation [56], MEGAHIT, Meraga (MEGAHIT combined with Meraculous [57]) and Minia [47] recovered the most substantial fraction of all genomes when compared to Ray Meta [36], Velvet [58] and OperaMS Scaffolder [56]. Although these analyses are useful, readers should keep in mind that only selected assemblers were compared. For example, the very popular high-performance metaSPAdes assembler was included in the CAMI comparison mentioned above. Besides this advice, the list of available assemblers continues to grow with new and promising tools that aim to improve assembly quality. One of these is the recently developed OPERA-MS assembler that allows hybrid assemblies of short reads together with long reads obtained from new technologies such as Nanopore sequencing [59]. Furthermore, tools to test different assemblers and parameter combinations provided by specific pipelines that integrate several of these methods, such as MetAMOS [60], are available. Similar approaches are offered by MeGAMerge [61] and GAM-NGS [62], which were developed to try multiple assemblers on the same data or to improve individual results by merging them. Once the assembly is obtained, its quality can be assessed using MetaQUAST [63], a tool that evaluates and compares metagenome assemblies based on alignments to close references.

### From pieces to taxa: binning

The contigs obtained after assembly can be used directly for gene prediction and functional assignment or for binning. Binning is the process of gathering the reads/contigs that belong to the same biological taxon (species, subspecies, or genus), and of classifying the resulting bins taxonomically. Although bins are used for taxonomic classification, they can also be analysed further for functional characterization. There are two main binning methods: taxonomy-dependent methods (also called supervised methods) and taxonomy-independent methods (also called unsupervised methods) (Fig. 1). Methods belonging to the first category, such as CARMA3 [64], MetaPhyler [65], or SOrt-ITEMS [66], use known reference genomes to map the contigs and are based on aligning metagenomic sequences to a reference. Other supervised methods are composition-based, and these rely on characteristics that can be extracted directly from the nucleotide sequences (e.g. oligonucleotide frequencies, GC content). Examples are TACOA [67], PhyloPythiaS+ [68], or Phymm [63]. The third subcategory of supervised methods (e.g. PhymmBL, a combination of Phymm and blast [63]) is based on a hybrid approach that is alignment- and composition-based, but their use has gradually declined. Some pipelines, such as IMG/M v.5.0 [69], MG-RAST v.4 [70] and MEGAN6 [71], integrate similarity-based binning algorithms. One of the drawbacks of supervised methods is the limited number of available sequenced genomes in the current databases and the long computing time necessary for aligning contigs to a reference.

Unsupervised methods do not have these limitations, since they do not rely on a reference genome. Hence, they have become more popular, and a panoply of such tools has been developed. According to the strategy used for binning [72], these can be classified into three main groups: nucleotide composition-based, abundance-based and hybrid methods. Nucleotide composition-based methods assume that the oligonucleotide composition of fragments from the same genome is more similar than that of different genomes [73, 74]. Examples are Metawatt [75], SCIMM [76], or LikelyBin [77]. The main problem with these methods is that their reliability depends on the read/contig length. DNA fragments that are too short will not provide enough information to extract the correct oligonucleotide frequency. Moreover, these methods generally do not manage to separate at a high taxonomy level, since they have difficulty in separating genomes with similar composition. Another concern is that species with low abundance can easily be misclassified into a larger bin belonging to highly abundant species [72]. This can be avoided by using the second category of abundance-based methods, which assumes that contigs that belong to the same genome should have similar abundance in the same sample. The methods using this assumption are AbundanceBin [78], which considers that reads are sampled from genomes following a Poisson distribution, or MBBC [79], which is also based on a Poisson distribution and Markov models to refine initial bins. These tools work with one sample, whereas others, such as Canopy [80], can work with a series of metagenomic samples. Finally, hybrid methods combining both composition- and abundance-based approaches, such as MetaCluster4 [81] CompostBin [82], MaxBin2 [83], MetaBAT2 [84], CONCOCT [85] and COCACOLA [86], have also been developed. Hybrid methods combining both approaches have been shown to outperform methods using only one approach [72].

As with assembly methods, the range of available tools for binning is overwhelming, and choosing from among them is a daunting task. However, comparative studies among binning methods can guide the user to select a proper tool, but these have been developed more slowly than for assembly tools [72]. In 2014, the previously mentioned CAMI was created to evaluate methods in metagenomics independently, comprehensively and without bias [56]. The first results were published in 2017, including the comparison of nine binners: MyCC [87], MaxBin 2.0 [83], MetaBAT2 [84], Metawatt 3.5 [75], CONCOCT [85], PhyloPythiaS+ [68], Taxator-tk [88], MEGAN6 [71] and Kraken [89]. Metawatt 3.5 and MaxBin 2.0 appeared to recover the largest number of genomes with high purity and completeness. However, there is no guarantee that the best-performing binners on CAMI-analysed datasets are the most appropriate for another dataset. In addition, new tools integrating new and different clustering approaches for guiding binning continue to appear. Some examples are the recently developed BMC3 [90], MetaBMF [91] and Solidbin [92]. BMC3C is an unsupervised ensemble-clustering method based on codon usage, composition and coverage information. MetaBMF is a fast reference-free binning method that can be used for large-scale metagenomic applications and allows the binning of DNA fragments accurately at both species and strain level. Finally, SolidBin is a semi-supervised method that uses sequence feature similarity and/or additional biological information to construct the bins.

After binning, reads can be mapped back to the bins, and each bin can be reassembled, which can produce longer contigs if the binning is successful [93]. Then, several methods can be applied to check the quality of the final bin obtained. One of the most popular ones is CheckM [94], which provides information on both genome completeness and contamination by using lineage-specific single-copy marker genes and single-copy orthologues. When marker genes are missing, the genome is probably not complete, and if marker genes are present multiple times, it suggests contamination. However, the user has to be cautious, as this tool is based on the core genome, which tends to co-assemble properly, but if chimeric genomes are generated through the assembly, they may be correct when using this evaluation software. A tool that can evaluate metagenomic binners given known reference genomes is AMBER [95]. Evaluation of the same genome binning submissions previously used in the first CAMI challenge with AMBER proposes MaxBin2 [83] and MetaBAT2 [84] as the binning methods that perform the best, with the caveat that performance when using a small set of synthetic data may not be representative of performance with real data.

Finally, tools combining multiple binning algorithms for the curation of bin assignments have been developed, such as Binning-refiner [96], DAS Tool [97], MetaWRAP [98], or ICoVeR [99]. Binning-refiner extracts shared contigs between two sets of obtained bins, reducing the contamination level and increasing the total size of the genome bins. DAS Tool integrates predictions from multiple established binning tools selected by the user in the same assembly and uses a consensus approach to select a single set of non-redundant, high-quality bins. In contrast, MetaWRAP uses the output bin sets of MetaBAT2 [84], MaxBin2 [83] and CONCOCT [85] to generate hybrid bin sets. Based on these, bin reassembly, read extraction from a given bin and assembly separate from the rest of the metagenome is carried out. Finally, ICoVeR allows the visualization of different binning results and their further supervised refinement. Other exciting tools for binning are pipelines such as Autometa [100], developed to separate microbial genomes from host genomes and other eukaryotic contaminants by using sequence homology, nucleotide composition, coverage and the presence of single-copy marker genes. Once filtered for contamination and completeness, the resulting bins are known as MAGs [101], in contrast to single amplified genomes (SAGs).

### Identification of coding regions: annotation

After the classification of sequences in taxonomic bins, the next step is to identify and annotate genes and regulatory elements. The MetaGene [102] gene-finding program was the first one designed to predict genes from fragmented genomic sequences. It uses the GC content of genome fragments to approximate codon frequencies and estimate the original codon usage of the genome, allowing one to predict genes. MetageneAnnotator is an upgraded version of MetaGene adapted for metagenomic data [103]. It allows the prediction of typical prokaryotic genes, but also atypical genes, such as horizontally transferred and prophage genes, as well as new ribosomal binding sites. Later, the heuristic model integrated in MetageneAnnotator was improved by Zhu and colleagues [104] and implemented in the software GeneMark.hmm, which showed higher accuracy and was adapted for metagenomes (MetaGeneMark2). The last version of GeneMark, GeneMarkS-2 [105], uses a multimodel approach for the detection of both typical and atypical genes. It is not based on read length but on species-specific oligonucleotide usage patterns, an approach that is indeed improving the accuracy of prokaryotic gene predictions. Further, this new version identifies genome-wide features of transcription and translation mechanisms.

Several other resources for gene annotation that are classically used, such as Glimmer [106] and Prodigal [107], have also developed new versions that are applicable for metagenomic data, namely MetaProdigal [108] and Glimmer-MG [109]. MetaProdigal specializes in identifying translation sites and can identify sequences that use alternative genetic codes. Glimmer-MG carries out taxonomic classifications using Phymm [110] to find closer reference genomes that are used to train models for gene prediction, and the first annotations are done based on this result and then unsupervised clustering using SCIMM [76] is employed to complete the annotations. A gene predictor developed to identify genes directly from both genomes and short reads is FragGeneScan [111], a program that uses both sequencing error models and codon usage in a hidden Markov model for gene calling.

Several comparative studies have also been undertaken to help in selecting the best calling method. For example, Kelley and colleagues compared Glimmer-MG, MetaGeneAnnotator and MetaGeneMark [109], and concluded that although it is computationally more demanding, Glimmer-MG shows the best performance for simulated metagenomes. Even using error-prone sequences, GlimmerM outperformed FragGeneScan and MetaGeneMark. Another study that compared GeneMark, Orphelia and Metagene-Annotator [112] concluded that for 100–400 bp sequence fragments, the best results were obtained when using a combination of all the methods, while GeneMark and Orphelia showed the best performance for 500 bp and longer sequences. Finally, a more recent comparison between FragGeneScan, MetaGeneAnnotator, MetaGeneMark, Orphelia and Prodigal found that FragGeneScan is better for calling genes in error-containing fragments, while Prodigal, MetaGeneAnnotator and MetaGeneMark are better suited for higher-quality sequences, such as assembled contigs [113]. Despite these comparative studies, the most currently used strategy and probably the best one to identify protein-coding genes uses a combination of different gene-calling tools, e.g. the JGI annotation pipeline [114], which uses GeneMark.hmm, MetaGeneAnnotator, Prodigal and FragGeneScan.

If one is interested in the eukaryotic sequences present in metagenomic samples, then gene calling is a more complex problem than in prokaryotes. GeneMark offers specific software for the annotation of eukaryotic genes, GeneMark.hmm-E and GeneMark.hmm-EIS (http://exon.gatech.edu/GeneMark/gmhmme.cgi) [115, 116]. In parallel, many tools that are specific for gene calling in eukaryotes have been developed, such us AUGUSTUS [117], Gnomon [118], or SNAP [119]. Others, such as EuGene [120], have been developed for both eukaryotic and prokaryotic genomes. Moreover, annotation pipelines such as MAKER2 [121] combine multiple annotation tools that run three different gene prediction programs (SNAP, GeneMark-ES and AUGUSTUS).

### Functional annotation and taxonomic profiling

Functional annotation of WGS metagenomics allows one to answer the question, which functional capacities are encoded in a microbial community? Once genome assembly, binning and gene calling have been done, many tools allow one to carry out functional annotations. The most common way to identify gene function is through similarity searches using classical tools such as blast. However, additional databases of broad scope such as Pfam [122], Interpro [123], PRIAM [124], or Metacyc [125] should be used to refine the predicted function. If instead of obtaining a broad functional overview, one is interested in identifying specific functions, specialized databases assembled for identifying e.g. metal detoxification genes, antibiotic resistance genes, or virulence factors can be used. These databases are generally more accurate and often contain manually curated sequence entries. Examples of well-curated databases are CARD [126] for antibiotic resistance genes or BacMet [127] for antibacterial biocide and metal resistance genes.

Since running all the tools mentioned above separately and integrating results for each gene from these different tools is not practical, integrated environments that group many of these methods, allowing automatic genome annotation, can be used. Among these, several online platforms support the submission of MAGs, such as MG-RAST v.4.0 [70], MicroScope [128], or IMG/M v.5.0 [69]. More recently, other, more flexible, tools that allow the processing of metagenomic data from raw data or contigs have been developed. One example is MOCAT2 [129], which allows quality trimming, decontamination, assembly, assembly revision and gene prediction. Another example is the previously mentioned assembly tool MetAMOS [60], which can also be used for taxonomic and functional annotation and validation and can be extended and custom-tailored to suit individual needs. The advantage of these pipelines is that they are straightforward to use. Hence, they also need to be used with caution. Oversimplification of the bioinformatics analysis of samples limits the possibilities as it offers less control of each step of the process and fewer parameters to choose from. Although very helpful, the main drawback lies in the fact that these pipelines have not been developed to make informed decisions at every step of the analysis process, as they essentially allow bioinformatic analysis without one being a bioinformatician [130]. As part of their simplified approach, the same parameters are often used for all analysed data. This is not an issue when the aim is to obtain a general overview of the community functions, but it can result in highly inaccurate annotations when one is searching for specific functions. In this case, the best choice is to use specific curated databases, as mentioned above.

Other options are standalone pipelines for the annotation of assembled contigs, scaffolds, or whole-genome sequences. Among these, we have the popular Prokka annotation tool [131], DFAST [132], which is especially useful to transfer annotations from other genomes, or the National Center for Biotechnology Information (NCBI) tool, PGAP [133]. For eukaryotes, there are also specific tools for functional annotation, such as the previously mentioned MAKER2, or the Genome Sequence Annotation Server (GenSAS) [134], which has recently become available. However, none of the previously mentioned tools has been developed specifically to cope with the typical problems associated with MAGs, such as poor quality assembly, possible contamination with foreign genes, or lack of close reference genomes. Recently developed tools such as the MetaErg [135] pipeline address these challenges, although at the cost of greater running time and increased computational resources. Going even further, the web application METAREP [136] allows one to analyse and compare annotated metagenomics datasets providing graphical summaries for top taxonomic and functional classifications, as well as a GO, NCBI Taxonomy and KEGG pathway browser.

Finally, catalogues of reference genes from different microbiomes are emerging and are becoming crucial for functional metagenomic analyses. Mapping of sequencing reads against theses catalogues allows taxonomic resolution of gene entries, together with linking of genes to MAGs and reconstructed full-length 16S rRNA genes [137]. For example, the pipeline MGS-Fast uses the reference catalogue of the human gut microbiome [138]. Although still incomplete, these existing catalogues continue to grow to become a detailed classification of the composition of each microbial ecosystem. Examples are the recent expansion of the human gut microbiome catalogue [139], improving the classification of understudied African and South American samples, or the reconstruction of microbial genomes from different human body cavities from Westernized versus non-Westernized populations [140].

The tools mentioned above allow us to decipher the functions associated with a particular metagenomic sample that has been processed using WGS. However, these data also have the potential to reveal which organism encodes these functions, although this question is generally answered using marker gene profiling, an approach that will be described extensively later in this review. The advantage of using WGS metagenomics is that it bypasses the biases that may be introduced during the PCR amplification of the marker gene. For this purpose, software such as the previously mentioned MEGAN6 tool [71], some of the previously described binning methods, e.g*.* DIAMOND [141], or some of the above-cited annotation pipelines, such as MG-RAST v.4 [142], can be used. Another recently developed tool is the MAGpy pipeline [143], which can identify the likely taxonomy of hundreds or thousands of MAGs, draw a taxonomic tree and carry out genome annotation. However, one major drawback associated with taxonomic profiling of MAGs is that generally strain differentiation is not possible, since MAGs represent aggregates of multiple similar strains [144]. This is a problem because sequencing studies of (opportunistic) pathogens have demonstrated that many microbial phenotypes are strain-specific [145].

Consequently, methods have recently been developed to also allow genome profiling of MAGs at the strain level. In particular, Segata and colleagues developed tools to profile strains accurately from metagenomes and scale strain profiling to many thousands of metagenomes with manually curated metadata such as MetaMLST [146] or StrainPhlAn and PanPhlAn [147]. Other similar tools are DESMAN [144] and MetaSVN [148]. DESMAN is a pipeline that solves the strain-level variation in MAGs in terms of nucleotide variation on core genes and the variation in gene complement, without the need for any reference genome. On the other hand, MetaSVN [148] calls SNVs on metagenomes mapped against reference genomes to estimate allele frequencies. Future research in this direction will be crucial to exploit the full potential of shotgun metagenomics in the fields of medicine, ecology and microbiology.

### The assembly-free approach

Assembled genomes have clear advantages for further functional analyses. However, to obtain correct assemblies when working with metagenomic samples remains a challenging task. This is because of the presence of genomic repeats, short overlap lengths, phylogenetically close organisms that can lead to false-positive alignment outputs of the assembly, joining non-overlapping fragments of two different parts of the genome or by producing chimeric contigs from different organisms. In the final assembly, genomic regions may be missing, and the quality of the assembly may be affected by factors such as genome size, sequencing technology, sequence length and coverage depth. Based on this, genome assembly has to be done and analysed with caution when performing WGS, and most of the reads obtained from these samples will remain non-assembled. Thus, software that allows the analysis of raw metagenomic data directly for both taxonomic classification and functional assignments has been developed.

The advantage of using WGS reads for taxonomic classification is that it allows the detection of organisms across all domains of life and alleviates biases due to primer choices for marker gene analyses [93]. The above-mentioned binning methods can be used in addition to, for example, MG-RASTv.4 [70], MEGAN6 [71], CARMA3 [64], or Taxator-tk [88], which are well-established tools for reference-based classification. These methods are highly accurate but have the disadvantage of being slow. To speed up the process, alternative methods such as Kraken [89] or Clark [149] replace the direct alignment of a query against a reference database by a fast-lookup method of fixed-length k-mers extracted from the query. Subsequent matching of the query k-mers to an index structure prebuilt from the references allows quick classification. The recent version of Kraken, Kraken2 [150], achieved a major reduction in memory usage. Alternatively, there are web-based tools such as Taxonomer [151]. Another fast tool is Centrifuge [152], an approach that reduces the high memory requirements of k-mer-indexing structures using a highly compressed Burrows–Wheeler-transformed Ferragina–Manzini (FM) index. This tool also implements a feature that combines shared sequences from closely related genomes, greatly reducing redundancy for species where dozens of strains have been sequenced. Another tool based on the FM index is Kaiju software [153], which uses a database of translated proteins, and the six-frame translations of reads are aligned against these protein databases. A more recently developed pipeline, taxMaps [154], reaches a classification accuracy that approximates that of blastn, and that is more precise than Centrifuge or Kaiju. Another possibility is reference-free methods such as PhylopythiaS+ [68] or Phymm and PhymmBL [110]. However, these are slower and often require relatively long query sequences in order to achieve sufficient entropy on the composition feature they use to classify [155]. A faster alternative is MetaPhlAn [156], with a recent new version, MetaPhlAn2 [157], which provides eukaryotic and viral quantitation. Again, the availability of software for this purpose is overwhelming for the user, and again comparative studies as preformed recently by Ye and colleagues [158] help to guide the choice for the subsequent analysis.

When working directly with reads for annotation, traditional tools such as blast are often too slow because of the significant amount of data being processed. Thus, new methods employing optimized strategies that allow comparison of nucleotide sequences to protein databases have been developed to speed up the process. The first tools were Usearch [159], BLAT5 [160] and the faster RAPSearch2 [161]. Since the quantity of data continues to increase, new tools such as DIAMOND [141], software that replaces blastx by reaching similar sensitivity levels but that is thousands of times faster by using double indexing, have been developed. Recently, new, even faster methods, such as PALADIN [162], have been developed to cope better with the analysis of the ever-increasing quantities of sequences. PALADIN provides results seven times faster than DIAMOND or GRASP2 [163]. Simultaneously, several pipelines were set up that integrate some of these and other previously mentioned tools, to allow direct annotation from raw sequencing data or contigs. For example, FUN4ME [164] integrates three tools: FragGeneScan for gene calling, RAPSearch2 for homology research and the MinPath [165] tool to allow biological pathway reconstruction. Classical, previously mentioned software such as MOCAT2 [129], the web portal MG-RAST v.4 [70], or the IMG/M v.5.0 [69] annotation server, also allow the comparison of metagenomic sequence reads to a reference database of functionally annotated protein families and use homology inference to annotate them. The more recent pipeline, MGS-Fast [166], allows both functional annotation and taxonomic profiling from reads and contigs. More recently, advanced tools such as HUMAnN2 [167] allow the inference of the functional and metabolic potential of a microbial metagenome directly from short sequence reads. The recently developed Carnelian [168] tool is recommended to perform comparative functional metagenomics. Further, more flexible tools enabling customizable annotation, such as MetaStorm [169], a web server that supports read or assembly annotation based on a reference dataset uploaded by the users, have been developed. This also provides enhanced interactive visualization and outperforms previous tools. The pipeline Shot-MAP [170] offers even more flexibility than MetaStorm. It was developed based on simulations to optimize metagenome annotation and allows users to select settings according to their data. For example, users can select from a variety of gene prediction and alignment algorithms, tune the specific thresholds used to classify reads into families or change the mapping parameters according to read length.

Many excellent tools exist for the analysis of metagenomic sequence data to learn about the diversity of the community and assemble the genomes that are present and functionally annotate them. However, the best results are obtained when different methods are combined, as proposed for integrated tools. Finally, metagenomic analyses have the potential to describe the microbial community present in a sample completely, including eukaryotes, prokaryotes and viruses. However, most of the tools described here cannot be used for viral sequences, but their analysis requires specific methods that are beyond the scope of this review (for a review, see Simmonds *et al*. [171, 172]).

## Marker gene analyses

Marker genes are conserved genes containing one or more hypervariable regions, which allow one to discriminate between different lineages. Since the discovery of its potential, rRNA genes, in particular, the 16S rRNA and 18S rRNA for bacteria and eukaryotes, respectively, are considered to be some of the best marker genes for studying phylogenetic relationships [173, 174]. Moreover, the ITS regions have been accepted as a gold standard to study fungi [3]. Several methodologies and software packages have been developed to improve the analysis of high-throughput sequencing data outputs due to the growing interest and importance of the analyses of biodiversity in microbial ecology. In this section, we present an update on the processing of marker gene data as well as best practice and the current challenges in this field. (The main steps are summarized in Fig. 1.)

### Operational taxonomic units (OTUs) and amplicon sequence variants (ASV) to perform diversity analyses

Operational taxonomic units (OTUs) have been chosen for microbial ecology research. OTUs that have 97 % identity are considered to be roughly approximate to ‘species’. In recent decades, OTUs have been the basis of many marker gene studies to characterize a large number of different microbial communities. They have been used for analysing soil [175], water [176, 177], or host-associated microbiomes [178–180]. However, OTU-based approaches also have certain disadvantages: (1) OTUs with 97 % identity are not necessarily equivalent to species level; (2) generally the number of estimated OTUs is higher than the real number of species due to sequencing errors; (3) OTUs are not sensitive enough to detect small variations between reads, and thus do not allow us to discriminate between closely related but different taxa. Therefore, recently, non-OTU-based methods such as DADA2 [8], Deblur [10], or UNOISE3 [9] have been developed to determine exact features named amplicon sequence variants (ASVs). These programs allow us to analyse the microbial diversity of various environments, such as the gut [181–183], oral [184], plant [185], or water microbiomes [186].

DADA2 is an open-source R package that models and corrects errors produced during Illumina sequencing, identifies ASVs and resolves differences of as little as one nucleotide [8]. The method is based on the Divisive Amplicon Denoising Algorithm (DADA), a model-based approach for correcting amplicon errors [187]. DADA2 performs filtering, dereplication, removal of singletons, sample inference, chimera identification and merging of paired-end reads, providing data that are ready for further ecological and statistical analysis. Deblur is a novel sub-operational-taxonomic-unit (sOTU) approach that estimates putative error-free sequences at a single-nucleotide resolution from Illumina sequencing, based on error profiles [10]. Deblur is computationally faster than other OTU methods and it shows similar or better sensitivity. Both DADA2 and Deblur are implemented in the QIIME 2 project [188]. In contrast to Deblur and DADA2, UNOISE3 does not depend on quality scores but on the one-pass clustering strategy that is based on two parameters with preset values that are curated to generate what the author called ‘zero-radius OTUs’ (the equivalent of ASVs) [9]. UNOISE3 is the most computationally efficient package, but is less accurate than either Deblur or DADA2. However, there will always be a trade-off between speed and accuracy in noise removal methods.

A comparison of the three denoising methods and the classic open-reference OTU clustering has shown that when using recommended settings for each pipeline, a similar community structure is found (close beta diversity values) (Box 1) [189]. The authors showed that DADA2 detects more ASVs than the other two denoising methods, suggesting that it could be useful in detecting micro-organisms from ‘the rare biosphere’; however, a higher rate of false positives accompanies this advantage. On the other hand, with the open-reference method, the number of OTUs is much higher than expected, since OTU-based methods tend to overestimate diversity [24]. The two methods can be considered to be equivalent for estimating beta diversity (Box 1) (comparisons of OTUs or ASVs based on relative abundances), but they give different outputs for alpha diversity (Box 1), in particular for low-abundance micro-organisms [189].

BOX 1. **Definitions and terms used in microbial ecology in the area of sequencing**

**Alpha diversity**: the diversity measured within a particular ecosystem or sample. It is commonly characterized by OTU/ASV richness, evenness and phylogenetic diversity. **Beta diversity**: diversity comparison between particular ecosystems or samples. It is commonly analysed through ecological and phylogenetic distances estimated from the sample composition. **Rarefaction curves**: plots representing the number of samples on the *x*-axis and the number of ‘species’ or diversity variants on the *y*-axis. The curves show how the richness increases with the increase of sequencing depth, and they are commonly used to select a threshold value (plateau) to perform diversity analyses. **Metadata**: all the variables and data relevant for the study providing information about the samples included. For example, in a theoretical study of a water-associated microbial community, typical metadata could be measures of temperature, pH, salinity, or oxygen levels from the samples analysed.

Since significant variations are associated with distinct methods, it is better not to compare results obtained from different pipelines. There is not ‘a universal method’, but choosing the best method depends on the type of data and the question to answer. For example, to compare samples enriched in phylogenetically related species requiring a high resolution (very similar sequences), ASV-based methods are more appropriate than OTU-based methods, since they detect single-nucleotide differences. However, ASV estimation methods have some limitations. For example, a single genome can contain multiple ASVs that can differ in more than one nucleotide, which may lead to erroneous taxonomic annotations. Thus, when genomic heterogeneity is essential in the study, it is better to choose a more conservative approach, such as the OTU-based methods [8]. Further, ASV methods are strongly affected by the quality of the data, and PCR errors during library preparation steps lead to the depletion of a large amount of useful information. When the quality of the data is not high enough, it is a more robust option to use an OTU-based approach. Once the method is chosen, the best practice is to adjust the parameter settings to the data analysed as much as possible to obtain the most accurate results.

### Taxonomic annotation and reference databases

Assigning taxonomy to OTUs/ASVs is a critical step in microbial community analyses since it answers the question, ‘who is there?’. Different classification methods and databases have been developed for the taxonomic assignment of the most common marker genes, the 16S rRNA and 18S RNA genes, and the ITS region.

Typical classification software is based on different algorithms, such as RDP classifier [190], UCLUST [159], or blast [191], coupled to reference databases. RDP classifier, one of the most applied tools in taxonomic assignments, allows classification at the genus level with an accuracy of around 80 % by using trained naive Bayes models [190]. On the other hand, UCLUST and blast are based on alignment methods [159, 191]. UCLUST, a clustering method that employs the USEARCH algorithm to assign sequences to clusters shows high sensitivity and is faster than blast. As part of the QIIME 2 project, the q2-feature-classifier, a taxonomic classification method based on novel machine learning and alignment-based methods, was developed [192]. The q2-feature-classifier provides two alignment-based taxonomy classifiers based on blast+ [193] and VSEARCH [194], and a multinomial naive Bayes machine learning classifier. Bokulich and colleagues performed a comparison of these novel methods with previous ones, including RDP classifier, UCLUST and blast. The new naive Bayes, VSEARCH and blast+ classifiers (included in the q2-feature-classifier plug-in) perform equally well or better than the previous generation of methods. Importantly, the optimization of parameter settings is as critical as choosing the correct method, because the accuracy of the classification and the ability to detect novel taxa may vary considerably depending on these choices [192]. Furthermore, the output is, of course, influenced by the databases, and thus they also need to be chosen carefully.

The primary databases used for 16S rRNA gene analyses are Greengenes [195], the Ribosomal Database Project [196] and silva [197]. silva also includes small and large subunits of the rRNA gene (16S/18S and 23S/28S). These curated databases are often integrated into the most common pipelines for marker gene analyses, such as QIIME 2 [188], mothur [198], or RDP classifier [190]. Micro-eukaryotic diversity has been characterized less than prokaryotic diversity, but it is essential to curate and unify all the available information into comprehensive databases. Besides silva, for the 18S rRNA gene, eukaryotic databases have been designed, such as EukRef [199], or a more group-specific database such as the Protist Ribosomal Reference database (PR^2^) [200] and the Planktonic foraminifera Ribosomal Reference database (PFR^2^) [201]. The EukRef project aims to improve the taxonomic information for eukaryotes based on 18S rRNA data, to associate it with their environmental metadata and to create better reference databases for amplicon studies [202]. Protists are a heterogenic group of organisms with a broad distribution and a high level of genetic and ecological diversity, making it difficult to infer their phylogeny and classification. To improve this, PR^2^, an 18S rRNA curated database focused on protists, was constructed. It includes other eukaryotes, such as metazoa, land plants, macrosporic fungi and eukaryotic organelles (mitochondrion, plastid) [200]. In contrast, PFR^2^ is a curated database of 18S rRNA from planktonic protists [201].

The most up-to-date databases to analyse fungal diversity based on the ITS marker region are UNITE [203] and the Warcup ITS training set [204]. Analysis of the ITS is not as straightforward as that for 16S rRNA or 18S rRNA, since the ITS region is highly variable in sequence and length, making it challenging to determine phylogenetic relationships. Warcup ITS is an ITS-derived training set adapted for use with the RDP classifier for the identification of fungi. The UNITE database can be handled from QIIME 2 [188], mothur [198] and the RDP classifier [190]. It is worth mentioning that databases are not perfect, since they contain sequencing and PCR errors, as well as incorrect sequence labels that may lead to wrong classifications. Further, there is a bias in databases towards human-associated pathogens, making the classification of other environments where micro-organisms are not associated with disease more difficult [205]. Classifications can be improved in those cases where the composition of a particular habitat is known (e.g. he human intestinal microbiome), and it is possible to create a personalized reference database including the resident micro-organisms [205, 206].

### From marker gene taxonomy to genetic functions

A primary difference between the the whole genome sequencing and marker gene approaches is that the latter does not give information about the functional capabilities associated with the taxa, although some approaches have been developed to link marker genes with their corresponding functional profiles [207–210]. One of these methods predicts the ‘metagenome’ from the marker gene data. Okuda and colleagues developed a method where 16S rRNA gene sequences obtained from denaturing gradient gel electrophoresis analysis (DGGE) are mapped to fully sequenced genomes to reconstruct virtual metagenomes [207]. Tax4Fun, phylogenetic investigation of communities by reconstruction of unobserved states (PICRUSt) and pangenome-based functional profiles for microbial communities (PanFP) predict functional profiles from 16S rRNA sequencing data [208–210]. Recently, with the update of PiCRUSt to PICRUSt2, the potential functions derived from 18S rRNA and ITS can also be inferred [211] from whole-genome sequencing metagenomics. The estimation of the functions present in a metagenome predicted from marker gene analyses depends on the availability of closely related reference genomes in databases or on the similarity of the 16S rRNA region. Since certain related micro-organisms have similar rRNA sequences but different genomic features, this can be a difficult task. In particular, incomplete functional profiles are obtained from samples enriched in novel species with no available genomes. Thus, these functional predictions are helpful, but they cannot be considered to be substitutes for whole genome-sequencing metagenomics.

### Normalization of marker gene-derived data

After the prediction of OTUs/ASVs, the starting point for ecological and statistical analyses is the establishment of an abundance table that reports the number of reads for each OTU/ASV per sample in columns and the OTU/ASV definitions in rows. This abundance table can also be based on the taxonomic information at specific levels (e.g. genus, family) depending on the purpose of the study. The data inform us about the differential sequencing effort between samples and the diversity of microbial communities. Abundance tables in the field of microbial communities are characterized by (i) different numbers of reads between samples and (ii) a high number of single variants (reported as ‘zero’ values in the abundance table) [212]. Since a sample only represents a fraction of the original microbial community, the analyses have to be performed as relative abundances. From the abundance table proportions of reads are estimated (the number of reads corresponds to each OTU/ASV/taxon divided by the number of reads of the sample). Relative abundance tables that sum to 1 and are non-negative are considered ‘compositional data’ and cannot be analysed with standard statistical approaches [213]. The variability in the number of sequences obtained per sample is due to sequencing factors such as effort, bias, or library preparation inaccuracies. A high abundance of ‘zero’ values, also known as sparsity, is usually due to (i) features that have a low abundance (such as rare species) and are not detected in samples where the sequencing effort is low; (ii) features that are unique for a sample or a group. Irregular sampling depth, sparsity and the compositional nature of the data are critical factors influencing the alpha and beta diversity results (Box 1). Thus, before performing ecological and diversity estimations, and statistical comparisons, it is crucial to normalize the data to obtain comparable samples and meaningful results [214]. The most used normalization method in microbial community studies is rarefaction of the abundance table to the same depth. The depth can be determined by choosing the values where the rarefaction curves reach the plateau (Box 1). In this situation, all the samples are rarefied and set to the same number of reads. However, this method has some disadvantages, since using a threshold for the number of reads might lead to a bias in diversity estimations. It can also imply a loss of OTUs/ASVs/taxa and (or) samples from the dataset due to the differential sequencing effort between samples.

The second group of methods is based on scaling the data. The estimation of the relative abundance table is already a normalization process known as total sum scaling (TSS). However, to deal with the compositional nature of the data, a promising group of scaling methods based on log-ratio transformations has been developed. A simple scaling method, the log upper quartile (logUQ), basically scales each sample by the 75th percentile of the distribution of counts before log transformation [215]. Paulson and colleagues developed the cumulative sum scaling (CSS) method implemented in the metagenomeSeq package [212]. CSS works with a scaling factor that is a fixed quantile derived from the OTU counts. Another method named common sum scaling (COM), divides the counts scaled to the minimum depth of each sample [216]. Some statistical methods are based on using centred log-ratios (CLRs) to normalize the data before comparisons or inferences are performed, for example SPIEC-EASI [217]. DESeq and edgeR are two methods that were initially developed to compare differential expression between genes and are implemented in the Bioconductor package (R software) [218, 219]. Both approaches have been adapted for microbial community studies. They involve a complex scaling transformation, including the relative log expression (RLE) method. Further, DESeq includes the variance stabilizing transformation (VST), while edgeR includes the trimmed mean of M-values (TMM).

Recent studies have analysed how normalization methods influence the output of standard analyses in microbial ecology studies (mainly from 16S rRNA data). A study comparing rarefaction to various scaling methods (logUQ, CSS, DESeqVS, edgeR-TMM) showed that either one or the other type could perform well in combination with specific beta diversity estimations [214]. For example, rarefaction showed adjusted results when combined with unweighted distance metrics such as Jaccard and unweighted UniFrac. On the other hand, scaling methods worked well with weighted distance measures such as the weighted UniFrac. The authors also showed that, compared to rarefaction, scaling methods are more influenced by library size and produce artefacts. Moreover, Badri and colleagues evaluated the effect of different normalization methods in correlation analyses and showed that VST and CLR are better than TSS, CSS, COM and RLE for analysing compositional microbiome data [220]. This study also showed that the results of analyses based on correlation, such as clustering or network inference, depend on the normalization methodology applied. A different study evaluated the performance of normalization methods in terms of their capacity to identify differentially abundant genes, calculate unbiased *P*-values and control the false discovery rate (FDR) [221]. It showed that methods based on TMM and RLE had the highest performance. At the same time, when larger sample sizes were analysed, CSS is also a suitable option.

In conclusion, choosing normalization methods is not trivial. So far, there is no consensus about which approach shows the best performance. However, the best result is obtained from a trade-off between the data features (sample size, sequencing depth) and the normalization method, combined with the coupled ecological and statistical analyses.

### Correcting by copy number

In marker gene studies, one of the well-known biases is the variation in gene copy numbers between species [222]. For example, there is considerable variability among bacteria, where the 16S rRNA copy number ranges from 1 to 15 [223]. The OTU/ASV counts are biased towards those species with higher copy numbers. Different software exists to correct the copy number, such as rrnDB [224], Copyrighter [225] and functions implemented in the picante R package [226] and pplacer [227]. These approaches have so far only been developed to analyse 16S rRNA gene data, although the copy number of the 18S rRNA gene is also variable between species [228, 229], and thus this should be considered during analysis. Moreover, the ITS copy number is variable, since it depends on the copy number of ribosomal genes and thus should also be considered when analysing fungal diversity data. Recently, the accuracy of these methods in microbiome analyses was assessed and compared independently [230]. It was shown that copy number correction approaches are not accurate enough to be included in microbial community studies. The authors recommend excluding the copy number information in microbial community analyses except when the identified variants are sufficiently closely related to sequenced genomes or if there is a need for correct proportions of the OTUs/ASVs. As copy number methods depend on sequenced and annotated genomes that are included in databases, it is still an unsolved issue in microbial ecology.

### Marker gene pipelines

In addition to the possibility of analysing marker genes in a step-by-step way, there are pipelines that facilitate complete analysis, from quality filtering to statistical comparisons. The most commonly used pipelines are QIIME (now updated to QIIME 2) [188, 231], mothur [198], UPARSE [9], or MG-RAST [232]. Comparative studies of these pipelines showed that all of them perform well for 16S rRNA gene data, but QIIME and mothur provide a complete collection of methods, functions, analytic tools and documentation so far [205, 233]. For 18S rRNA and ITS data, fewer comparative studies between pipelines have been performed than for 16S rRNA. One of these studies showed that different pipelines, including QIIME and mothur, performed similarly for the 18S rRNA for analyses at high taxonomic levels and excluding the rare biosphere (taxon less abundant than 1%) [234]. A different study suggested as the best approach was to use not only one pipeline, but to combine tools from different pipelines, such as QIIME and mothur [235].

## Alpha and beta diversity

**Fig. 3.**
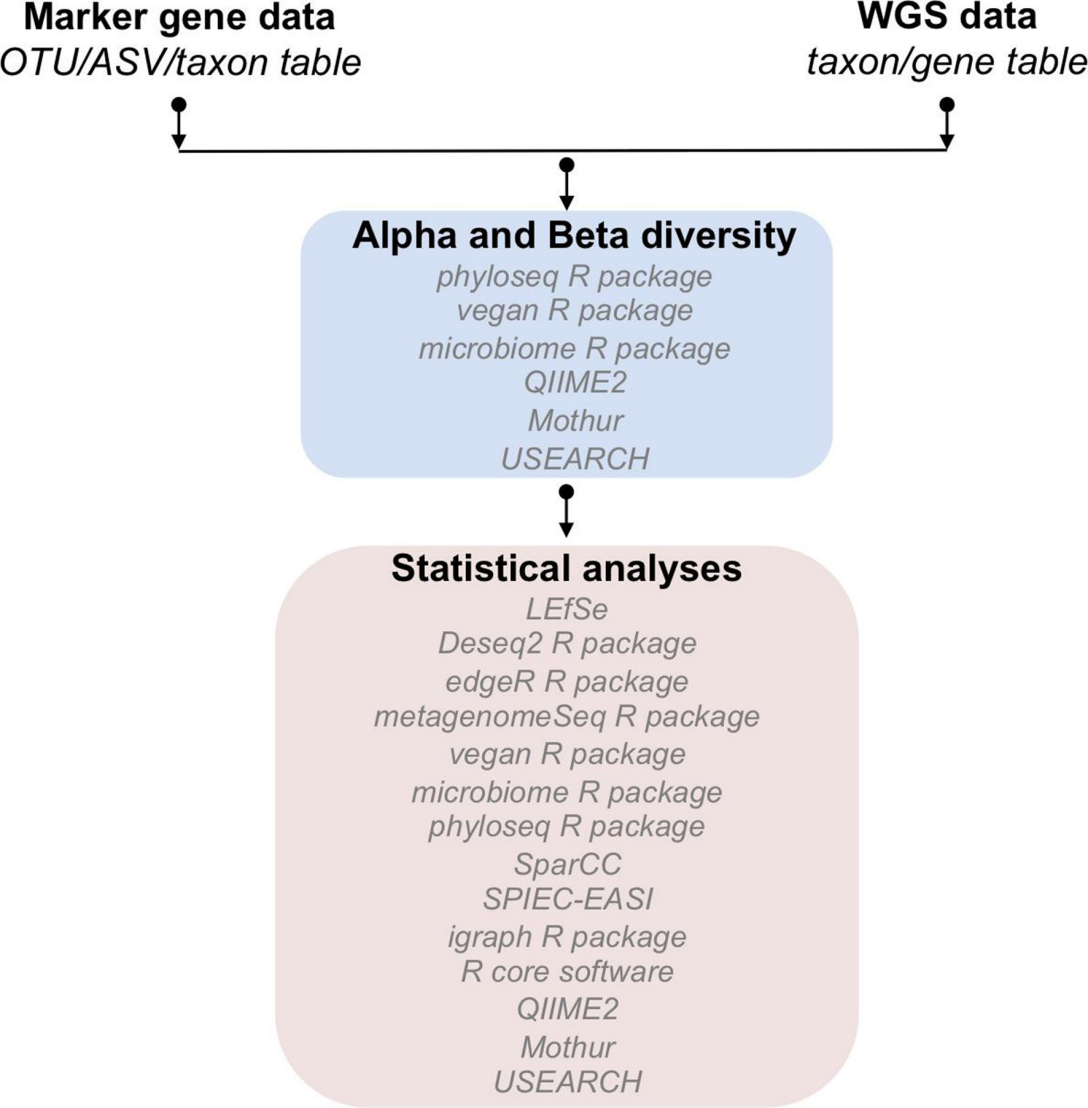
Summary of the software related to alpha and beta diversity analyses and the main statistical approaches for marker gene- and WGS-derived data.

In microbial ecology, diversity is typically described within (alpha diversity) or between (beta diversity) samples (Box 1). Most of these estimations can be applied to marker gene- and WGS-derived data (taxonomic composition and genes). A summary of the most common analyses is shown in Fig. 3. Alpha diversity quantifies diversity within samples and is generally characterized by variant richness (estimated with the Chao 1, number of OTUs/ASVs and Abundance Coverage-based Estimator: ACE) [236, 237]. These metrics estimate or count the number of variants, but they do not contemplate their abundance. Other metrics include the species richness and evenness, e.g. the Shannon or Simpson indexes [238, 239]. On the other hand, Faith’s phylogenetic diversity has been conceived to include the phylogenetic relationship in microbial diversity predictions [240] (Box 1). Two groups of different distances can be applied to compare the samples based on their composition (beta diversity): ‘non-phylogenetic’ distances such as Bray–Curtis [241] or Jaccard [242] and ‘phylogenetic’ ones such as unweighted and weighted UniFrac [243] (Box 1). Bray–Curtis and weighted Unifrac are quantitative, while Jaccard and unweighted Unifrac are qualitative.

Ordination techniques such as principal coordinate analysis (PCoA), canonical correspondence analysis (CCA), principal component analysis (PCA), or non-metric multidimensional scaling (NMDS) are practical exploratory approaches [20, 244, 245]. These techniques summarize the variability and help to identify patterns in the microbial composition of the samples. Classification methods are useful when groups of samples have already been classified and can be compared according to the metadata (e.g. disease status, diet). For example, it is possible to identify OTUs/ASVs/taxa/genes that explain the difference between the two groups (e.g. health or disease). On the other hand, clustering analyses allow us to identify clusters of samples in terms of OTU/ASV/taxon/gene composition (distance matrix). The distance and clustering algorithms influence the outcome of clustering analyses; thus, the identified clusters need to be confirmed by multiple methods [244, 246]. Combined with clustering analysis, heat maps are convenient to visualize the relative abundance of the OTUs/ASVs/taxa/genes, explaining the differences between the clusters. Calculation of meaningful metrics for alpha and beta diversity (Box 1) analyses can be performed with different software, including QIIME 2 [188], mothur [198], USEARCH [159] and the R software packages: phyloseq [247], microbiome [248] or vegan [249].

## A glance at the main statistical analyses

Statistical analyses of alpha and beta diversity data should be based on the biological question asked and the results of the exploratory analyses. Some of the most used statistical analyses for microbial ecology studies are shown in Fig. 3. A common question in microbial community research is whether there are statistically significant differences between two conditions (e.g. the water microbial community of two different geographical areas). To analyse whether the alpha-diversity (e.g. the Shannon index) differs between two groups, non-parametric tests such as the Wilcoxon rank-signed test [250] or the Kruskal–Wallis test [251] can be used for pairwise comparisons. Further, to identify if two groups have a statistically significant different composition (beta diversity), multivariate tests such as permutational multivariate analysis of variance (PERMANOVA) [252], analyses of similarities (ANOSIM) [253] or the Mantel test [254] are widely used in ecology. These non-parametric tests are more robust on marker gene data than traditional methods such as the Student’s *t*-test, analysis of variance (ANOVA), or multivariate analysis of variance (MANOVA). A comparative study examined the effects of heterogeneity of multivariate dispersions of the data on PERMANOVA, ANOSIM and Mantel test [255]. The authors showed that PERMANOVA performed better in detecting changes in community structure than the Mantel test, which performed better than ANOSIM. However, none of the tests was reliable when facing unbalanced designs [255]. To identify OTUs/ASVs/taxa/genes with significantly differential relative abundance between two conditions (as part of a biomarker identification), differential abundance tests have been developed [256, 257]. The linear discriminant analysis (LDA) effect size (LEfSe) focuses on biomarker identification from metagenomic data. The methodology applies standard tests for statistical significance combined with methods related to biological consistency and effect size [257].

The analysis of the composition of microbiomes (ANCOM) is a more recent test adjusted to the structure of microbial community structures, and it accounts for the compositional data [256]. Other methods also considering the compositional data effect are Deseq2 [258], edgeR [219], Voom [259], or metagenomeSeq [212]. Weiss and collaborators performed a comparison between some of these differential abundance tests, including the Wilcoxon rank-sum test, Deseq2, edgeR, Voom, metagenomeSeq and ANOSIM [214]. The authors proposed that tests based on general linear models using negative binomial or log-ratios would be useful. For example, Deseq2 works well on smaller datasets, but it shows a higher false discovery rate with a high number of samples, larger and/or uneven library sizes, and/or compositional effects. However, ANCOM is more stable in terms of false discovery rates for a wide range of sample sizes. Thus, as mentioned for the normalization procedure, it is vital to consider the structure of the data, the library and the sample size, since these factors affect the output directly when they are combined with the differential abundance tests.

Many studies on microbial communities incorporate network analyses to infer microbial ecological interactions and/or external variables [260–262]. These involve the identification of dependences between members of the microbial communities, generally based on correlation analyses. Where standard correlations such as Spearman’s rank correlation fail, some software packages adapted for marker gene data, such as SparCC [263] and SParse InversE Covariance Estimation for Ecological Association Inference (SPIEC-EASI) [217], can be applied. SparCC allows the inference of correlations between genes or species from microbial data considering the compositionality of the data [263]. SPIEC-EASI is a program that takes the compositional nature of marker gene data and a graphical model inference framework to infer possible microbial relations (tested with 16S rRNA data) into account. The output of both methods can be coupled with visualization programs such as the igraph package [264] implemented in R. This package can handle large graphs and can be embedded in other programming languages, such as Python, Perl, GNU and R, and can be used both interactively and non-interactively.

To test the significance of associations between environmental variables from the metadata (e.g. temperature, PH, oxygen) and microbial composition, multivariate ANOVA based on dissimilarities (Adonis) is widely used [249]. Recently, the microbial ecology research community has increasingly moved to machine learning techniques to make predictions and identify correlations and interactions between the microbial community composition and metadata variables [265]. Machine- earning-based approaches have been applied extensively to human microbiome studies (reviewed and compared by Zhou and Gallins [266]). Some of the most used methods are the ‘random forests’ that classify based on decision trees and ‘neural networks’ that are based on an interconnected and weighted feed-forward network of nodes to map inputs and outcomes [267–269]. Zhou and Gallins compared the performance of 17 datasets (microbiome) analysed with numerous prediction methods. The authors proposed that ‘decision tree’ methods and ‘neural networks’ performed well with the analysed datasets. It was also shown that previous OTU feature reduction of the data with the hierarchical feature engineering (HFE) algorithm [270] improved performance for most of the methods. In order to validate results obtained from machine learning approaches, The results obtained from machine learning approaches should be validated by applying other experimental and computational techniques, including independent cross-validation tests [245]. Some programs, including many of those discussed above, are R packages, including phyloseq [247], microbiome [248], vegan [249] and the R core, as well as pipelines such as QIIME 2 [188], mothur [198], or USEARCH [159].

## Conclusions

New tools are continuously emerging and evolving to adapt to sequencing technologies and metagenomic approaches. The most difficult issue for the user is definitively not to get lost among all possible choices. Indeed, the best approach is to compare the results obtained using several of the available tools. However, this is very time consuming and also requires one to acquire knowledge on how to use each tool. Finally, correct interpretation of the results obtained from the application of different analyses tools is necessary to form conclusions. Hence, when one is not making such comparisons using one’s own data, the comparative studies and reviews published regularly to guide the user are constructive and should be read and analysed carefully to guide one’s final decision on which tool to use.
